# Parental health literacy in parents of adolescents aged 16–19: a qualitative study

**DOI:** 10.1186/s12889-026-26471-9

**Published:** 2026-01-31

**Authors:** Hilde Timenes Mikkelsen, Tina Lien Barken, Gudrun Rohde, Sølvi Helseth, Siv Skarstein

**Affiliations:** 1 Department of Health and Nursing, Faculty of Health and Sport Sciences, University of Agder, Postbox 422, Kristiansand, 4604 Norway; 2 Department of Clinical Research, Sorlandet Hospital, Kristiansand, Norway; 3 Department of Nursing and Health Promotion, Faculty of Health Sciences, Oslo Metropolitan University, Oslo, Norway

**Keywords:** Parent, Adolescent, Late stage, Parental health literacy, Qualitative study

## Abstract

**Background:**

Parental health literacy is crucial for adolescent health outcomes — particularly during late adolescence, when teenagers take on greater health responsibility. There is a need for further research into parental health literacy and how parents access, understand, appraise, and apply health information, as this is vital for assisting their adolescents in making well-informed health decisions. This study aims to provide a deeper understanding of how parents of late-stage adolescents perceive their parental health literacy and to explore their experiences in transferring responsibility for health-related decisions to their adolescents.

**Methods:**

A study with a qualitative design was carried out using individual semi-structured interviews with 15 Norwegian parents of adolescents aged 16–19 years. Braun and Clarke’s reflexive thematic analysis was employed to analyze the data.

**Results:**

Three major themes were generated: (1) Transfer of health-related responsibility: from parental oversight to adolescent self-management, (2) parental health literacy and source credibility: navigating trustworthy information, and (3) enhancing parental health literacy to better support adolescents’ informed health decision‑making. Parents experience a shift as they balance their guidance with the adolescents’ growing autonomy. This involves selecting credible health information from digital platforms, healthcare professionals, and personal networks, underscoring the importance of parental health literacy in fostering adolescents’ informed decision-making and self-management of health.

**Conclusions:**

Parents of late‑stage adolescents are committed to supporting their children in managing health-related issues and to help develop adolescents’ capacity for autonomous health management, emphasizing the importance of parental health literacy. By facilitating the development of critical appraisal skills, and guiding informed decision-making, parents may contribute to strengthening adolescents’ competence and confidence in handling health-related matters more safely and independently. However, parents reported that losing access to their child’s health information after age 16 may hinder their ability to help. Empowering parents through strengthening their HL may improve individual and family health outcomes.

**Supplementary Information:**

The online version contains supplementary material available at 10.1186/s12889-026-26471-9.

## Background

Health literacy (HL) empowers individuals to act appropriately in new and changing health-related circumstances by utilizing advanced cognitive and social skills [1]. It is defined as the knowledge, motivation, and competencies to access, understand, appraise and apply health information for decisions regarding healthcare, disease prevention and health promotion [2]. These decisions encompass lifestyle choices, disease prevention measures, self-management of illness, and the use of healthcare services. Recognized as a social determinant of health [3], HL plays a crucial role in shaping health outcomes across populations. Nutbeam’s [4] framework delineates HL into three progressively advanced skill sets: functional literacy, involving health-related reading, writing, and numeracy; interactive/ communicative literacy, which includes cognitive and social skills needed for understanding and participating in health communication; and critical literacy, encompassing the skills required to act on health information for personal and social benefit.

Parents represent a particularly significant subgroup for public health and HL research [5]. As caregivers, they are responsible not only for their own health but also for the health and well-being of their children [6–8]. This significance becomes particularly evident during late adolescence, spanning ages 15–19 years. During this period, adolescents exhibit an increased drive for autonomy and independence, often distancing themselves from their parents [9–12]. This stage is foundational for future health, as adolescents become more accountable for their health behaviors [13]. Adolescents’ legal authority in health matters differs significantly, depending on clinical context, local legislation, cultural norms, and specific policy reforms. In Norway, adolescents gain legal authority over their health decisions when they reach the age of 16. This includes deciding whether to undergo treatment and determining who can be informed about their health status. Parents automatically lose access to their adolescents’ health information at this point [14]. Consequently, parents must navigate new aspects of their children’s independence and potential risk behaviors. Research has indicated that striking the right balance between providing support and allowing adolescents to assume more responsibility can be challenging for parents [15–18].

Our understanding of parental HL in this study is based on an established definition of health literacy [2]; we define parental HL as parents’ knowledge, motivation, and competencies to access, understand, appraise, and apply health information to make evidence-based decisions related to their child’s health. Parental HL plays a critical role in influencing adolescent health outcomes [8]. Studies have indicated that parents with high levels of education tend to excel in searching for and integrating health information, thereby benefiting their children’s HL [19]. The relationship between parental HL and children’s HL has been systematically reviewed, highlighting its significance [20]. Despite its importance, the understanding of parental HL during adolescence remains limited, especially concerning the decisions and choices parents make about their adolescents’ health and their experiences with transferring the responsibility for health-related decisions to their adolescents. It is essential to determine whether parents feel equipped with sufficient information to support their adolescents’ health and health behaviors. This includes understanding how well parents comprehend health information, the methods they use to obtain it, and their confidence in its credibility.

While most studies have focused on parents of adolescents within specific patient groups it is crucial to expand this knowledge to parents of adolescents in the community. Such an understanding can inform health promotion interventions and policy development. The aims of this study were (1) to provide a deeper understanding of how parents of late-stage adolescents perceive their parental HL and (2) to explore parents’ experiences in transferring the responsibility for health-related decisions to their adolescents. By exploring parents’ perceptions and experiences, the study seeks to identify potential areas for intervention and support, ultimately enhancing the ability of parents to access and utilize reliable health resources during this pivotal stage of their adolescents’ development.

## Methods

### Design

This study employed a qualitative design grounded in an interpretive, constructivist epistemology, which views knowledge as situated and context-dependent [21, 22] It adopted a hermeneutic orientation that emphasizes interpreting meaning and experience within social and cultural contexts [23]. Semi-structured individual interviews were conducted to explore the nuanced meanings and insights that parents associate with their ability to access, understand, appraise, and apply health information as they support their adolescents in navigating the complexities of transitioning toward greater independence. This approach allows us to delve deeply into the subjective realities of parental HL, providing a rich and comprehensive understanding of the challenges and opportunities inherent in their role during this critical developmental stage [24, 25].

### Participants and data collection

The study is part of the broader Norwegian mixed-method study titled “Start Young – Quality of Life and Pain in Generations” [26]. The present study constitutes a segment of the qualitative part of the Start Young study and was conducted from September 2023 to January 2024. A total of 15 parents of adolescents aged 16–19 years were included in the study. Participants were recruited using purposive sampling, a method in which the researcher chooses participants based on their alignment with the inclusion criteria [25]. Inclusion criteria were being a parent of at least one adolescent aged 16–19 years and the ability to speak Norwegian.

Participants were recruited via public posts on the social media platform Facebook and the snowball method, a nonprobability sampling technique employed when the target population is difficult to access. This approach allows participants who have already agreed to take part to inform other potential candidates about the study [27]. Five participants were recruited via Facebook, and ten participants were recruited via the snowball method. Interested individuals were directed to a dedicated web page for more information about the study [28]. All participants gave digitally signed consent on the study’s dedicated web page prior to participation, confirming that they understood the study and agreed to take part voluntarily. Consent was repeated and confirmed orally immediately before each interview.

An interview guide was developed for this study based on prior research and theory on the topic (Supplementary file 1). To gain a better understanding of the parents’ HL challenges and supportive resources, HL questions in the interview guide were inspired by the Conversational Health Literacy Assessment Tool developed by O’Hara et al. [29]. Because the tool examines everyday health‑information practices and decision‑making, it was considered well suited to our aim. Examples of questions are “How do you typically obtain information regarding health and illness?” and “Do you feel confident in the decisions you make regarding your child’s health?” The interview guide also covered aspects related to parental quality of life and the parental role during late-stage adolescence. Interview questions addressing HL informed the analyses reported here; questions addressing quality of life and the parental role are presented in a separate article that is currently under review.

Authors HTM, TLB, and SS conducted and transcribed the interviews. The interviews lasted between 30 and 45 min and were conducted using the digital platform Zoom. Each interview was audio‑recorded using an external digital recorder. No Zoom recordings or screenshots were taken. Participants were encouraged to discuss their experiences openly. Throughout the interviews, the interviewers ensured alignment between their interpretation and participants’ understanding using mirroring techniques and follow-up questions; Interviewers paraphrased participants’ statements, repeated key phrases, and summarized key points to invite participant’s confirmation or correction [25]. A digital transcription program named Autotekst [30] was partly used, supplemented by manual transcription.

### Analysis

The data were analyzed using reflexive thematic analysis, adhering to the six-step framework developed by Braun and Clarke [24], with emphasis on iterative and reflective interpretation. In step 1, three authors (HTM, TLB and SS) individually read and re‑read all transcripts, listened to recordings and made initial analytic notes before they in step 2 identified and systematically coded meaning units. Important codes and quotes were transferred into a table. In step 3, the three authors met to compare coding frames, examine underlying assumptions, and construct preliminary themes and subthemes. In step 4, the full author team convened for a collaborative meeting where authors HTM, TLB, and SS presented their initial insights and discussed the preliminary results with the other authors. The discussion was aimed at reaching a consensus on the themes generated, grounded in the reflexive nature of thematic analysis that encourages dialogue and the co-construction of meaning. The entire dataset was then reviewed once more to ensure that the preliminary theme names were logical and descriptive for the entire dataset. In step 5, we refined and defined each theme and subtheme to reflect their central meaning; theme labels were chosen to be clear, descriptive, and engaging. In instances of discrepancies, the original data were revisited, and interpretations negotiated until a shared understanding was achieved. In step 6, we wrote the presentation of results. While doing this, we repeatedly revisited steps 2–5, which led to thorough re‑examination of transcripts and coding. The authors jointly selected quotations that best reflected each theme.

The authors actively participated in the knowledge-making process and maintained reflexivity by attending to how analytical choices and theoretical positioning shaped their interpretation of parents’ descriptions of parental HL [24]. Consistent with a hermeneutic orientation, an iterative movement between part and whole ensured themes were rigorously developed before finalization. Analytic discussions examined how the authors’ assumptions, disciplinary backgrounds, and perspectives influenced coding and interpretation. Interviews and analysis were conducted by authors with professional experience in adolescent health as nurses and personal experience of parenting adolescents, which was acknowledged as a potential influence. To enhance reflexivity, the authors kept analytic memos, cross-checked coding, and discussed interpretations. The process was primarily data-driven, collaborative, and reflexive, and data were analyzed in line with Braun and Clarke [24], treating themes as analytic constructions rather than objective patterns and prioritizing meaning over frequency.

### Ethical considerations

This study carefully addressed ethical considerations to ensure the protection and respect of participants’ rights, following the principles outlined in the Helsinki Declaration [31]. Participants received clear information about the study’s aims, procedures, potential risks, and benefits. Informed consent was obtained digitally and orally, confirming participants’ voluntary participation and acknowledging their right to withdraw from the study at any time. Anonymization techniques ensured confidentiality. Identifying details were removed or replaced with participant codes during transcription; only minimal IDs (e.g., “P1”) appear in transcripts and quotes. The linkage key and any identifying records were subsequently destroyed. All data were stored on a secure, password‑protected institutional server with restricted access. No identifiable information was stored. At the start of each interview the interviewer emphasized that participants could freely express their views and decline to answer or skip any questions they found sensitive. No participants reported any questions as uncomfortable. All study procedures were approved by the Norwegian Centre for Research Data (Ref: 304463) and the board of ethics at the Faculty of Health and Sport Sciences at the University of Agder.

## Results

Fifteen parents, 14 mothers and one father, participated in the study. All were parents of at least one adolescent aged 16 to 19 years. The parents’ ages ranged from 41 to 56 years. Among these parents, 14 had higher education, 13 were employed, and two were receiving disability benefits. The thematic analysis generated three themes: (1) Transfer of health-related responsibility: from parental oversight to adolescent self-management, (2) parental HL and source credibility: navigating trustworthy information, and (3) enhancing parental HL to better support adolescents’ informed health decision‑making. Throughout the presentation of the results, quotes are consistently utilized to illustrate the findings. The parents are cited as P1, P2, and so on.

### Transfer of health-related responsibility: from parental oversight to adolescent self-management

The participants noted that the transition from childhood to adolescence introduces a gap between parents and adolescents, characterized by parents losing their comprehensive overview and feeling insufficiently prepared for the upcoming changes. Participants expressed a desire for clearer information regarding the legal changes at age 16 that affect parental insight into their children’s health information, advocating for both parents and adolescents to be better informed about this transition in advance. Several participants articulated the challenges they faced when their adolescents reached the age of 16; like this mom expressed it

Sometimes, they face challenges they might want to solve on their own. It’s easy to stand outside and see how it could have been resolved. Sometimes, you interfere too much or perhaps too little. It’s a process of trial and error—more so than when they were small. (P9)

A recurring theme in the interviews was that parents experienced a lack of information and support before and during this critical period regarding the legal changes that occur at age 16, which affect their parental role and access rights. Several parents expressed a desire for guidance on how to best navigate these changes. One participant shared: “When they turn 16 and I no longer have access to the health information; it is difficult to be able to follow up on any challenges they have” (P3). The uncertainty of where to draw the line between respecting adolescent privacy and ensuring parental involvement in serious health matters was a concern. One participant pondered,

Where is the limit for me to get … If it’s something serious, I hope I get information. So where do you draw the line for me to be informed, even if she doesn’t want me to? For now, I feel that we have an open and good dialogue. But I never know if she’ll suddenly become … If she suddenly became pregnant and had an abortion, would I have been informed when she’s 16? (P8)

This transition, which included transferring responsibility for health-related decisions to their adolescents, brought practical challenges, and the parents grappled with the reality that their adolescents now needed to manage health-related tasks independently.

### Parental health literacy and source credibility: navigating trustworthy information

Most participants expressed confidence in their parental HL, particularly in their ability to find trustworthy health information from reliable sources. Many participants had higher education, were occupationally active, and worked within the health sector, which contributed to their critical approach to sourcing information. One participant shared, “I feel that I am quite critical of sources, and that is natural through the job I have. So I relate to public pages and pages that I feel have high credibility” (P1). However, challenges arose when confronting new or complex topics, such as new vaccinations, for which research-based knowledge was limited. Some participants felt that this could hinder their ability to make informed decisions confidently and impair their efforts to prepare their adolescents for making informed health decisions.

Several participants said they were relying on trustworthy sources and trusting information provided by the public sector. To seek information regarding their adolescents, they frequently turned to various websites, general practitioners, or school health professionals. However, despite their confidence, some participants found it difficult to navigate the vast array of online sources and discern safe information. They struggled to distinguish between credible and unreliable online information. When uncertain, participants often reached out to healthcare providers or other reliable sources for further clarification.

Despite their efforts to convey healthy habits regarding sleep, nutrition, and other areas, all participants experienced that their adolescents often made independent choices that did not necessarily align with parental advice. The participants acknowledged their lack of expertise in understanding the impact of social media, societal pressures, and the expectations for success within the adolescents’ world. Hence, the parents felt the need to approach these topics wisely and carefully. As one participant noted, “It’s one thing to know [what is wise in terms of sleep and diet]; another thing is to be able to pass it on [to the adolescent]” (P6).

### Enhancing parental HL to better support adolescents’ informed health decision‑making

Participants in the study expressed a strong desire for comprehensive information to enhance their parental HL to help them guide their adolescents through the complexities of the teenage years. They emphasized the importance of this information in areas such as the impact of social media, societal pressures, mental health, sexual health, nutrition, substance use, vaccines, and digital literacy.

A desire to equip adolescents with the ability to critically assess information and make good choices regarding their health, impacting overall health, was highlighted. One participant remarked on the challenge of keeping up with information:

Parents need information about everything—from what’s going on in the adolescent’s body and brain to what’s happening out in society, including social media and such things. I find that difficult. It’s challenging to keep up with—to understand, to comprehend how it affects you. I notice that I’m constantly lagging behind. (P3)

Several parents mentioned a feeling of shame when facing challenges with their adolescents, noting that these issues differ significantly from those encountered with younger children. One participant stated,

It’s a bit difficult to talk to other parents about it if your adolescent is facing challenges, because it’s almost somewhat shameful. This is easier when the children are small, and you also have more settings where you meet others in the same situation. (P6)

Stress and pressure from societal expectations among adolescents emerged as a significant concern, and the participants acknowledged the importance of having the courage to set boundaries to safeguard their adolescents. Several participants spoke about the need to stand firm amid the turbulent emotions that adolescents experience, striving to be “good enough” to support their adolescents through the challenging times. As one participant remarked, “We need to understand the ups and downs of the teenage years, and we also need to receive guidance to withstand it” (P1). Participants acknowledged that the experiences and parenting strategies employed during early childhood have implications for the teenage phase, affecting how adolescents manage increasing autonomy and external pressures.

## Discussion

This study aimed to provide a deeper understanding of how parents of late-stage adolescents perceive their parental HL and their experiences with transferring the responsibility for health-related decisions to their adolescents. Based on the analysis of the interviews, the following three themes were generated: (1) Transfer of health-related responsibility: from parental oversight to adolescent self-management, (2) parental HL and source credibility: navigating trustworthy information, and (3) enhancing parental HL to better support adolescents’ informed health decision‑making. Our discussion will interpret the findings across the HL definition framework [2] to explore the nuanced meanings and insights that parents associate with their knowledge, motivation, and competencies to access, understand, appraise, and apply health information as they support their adolescents in managing the complexities of transitioning toward greater independence.

### Accessing trustworthy health information

The ability to access trustworthy health information is the first cornerstone of HL [2]. Parents in this study expressed confidence in locating health information from formal sources, such as general practitioners, school nurses, and public health websites. This aligns with prior research demonstrating that parents routinely turn to health professionals and institutional sites when seeking guidance on adolescent health [32]. However, our study revealed that challenges arose when confronting new or complex topics, such as new vaccinations, with limited research-based knowledge. This indicates that healthcare providers should strive to improve communication and create easy-to-access resources to help parents find accurate health information. Additionally, more investment in research can ensure quicker access to reliable knowledge, aiding informed decision-making.

Despite having high educational backgrounds and working within the health sector, many parents in this study found it challenging to navigate the overwhelming amount of digital content. This experience resonates with findings from Kubb and Foran [33], who identified that while parents globally seek online health information extensively, they are often overwhelmed by the volume and variability in credibility. The digital divide adds a layer of inequity. Estacio et al. [34] noted that access to online information is influenced by sociodemographic factors such as income, education, and digital skills. Thus, while this study’s sample seems to have high HL, broader parent populations may face greater barriers, requiring tailored digital HL interventions.

### Understanding health information

Although parents in this study felt capable of understanding general health advice, they reported difficulty in interpreting adolescent-specific issues, such as mental health, social media influences, and pressure from societal expectations. These themes underscore the generational gap in interpreting health behavior and societal trends, echoing concerns from previous studies that modern parenting requires constant adaptation to evolving environments [35, 36].

Several participants expressed confusion and concern about parents’ rights and responsibilities regarding adolescents’ health matters during late adolescence, highlighting a systemic gap in how the legal transition at age 16 is communicated and supported. Reduced access to health information may hinder parents’ ability to understand when interventions might be necessary. While this legal change fosters adolescent autonomy [14], our study indicates that many parents feel ill-prepared for it. As discussed by Speros et al. [1], understanding goes beyond comprehension; it involves functional literacy in context. If parents lack insight into how and when to act, their understanding remains incomplete. From the perspective of Nutbeam’s model [4], these findings suggest that parents may have shortcomings in interactive health literacy (limited access to and understanding of relevant information) and in critical health literacy (insufficient capacity to evaluate and respond to systemic issues), which together may hinder timely parental intervention. This indicates a need for clearer communication about legal transitions at age 16 and parental responsibility. However, discussing adolescents’ right to privacy versus parents’ responsibilities is an important topic for both family and societal discourse.

### Appraisal of health information

Appraisal involves the ability to critically evaluate health information for its credibility, accuracy, and personal relevance [2]. Even though many parents in this study considered themselves perceptive, often citing their professional background, they admitted to lack confidence in evaluating online content and information aimed at adolescents. Critical HL involves not only assessment but also the confidence to analyze information critically and utilize it in health decision-making. Our study suggests this capability may be compromised by information overload or the prevalence of misinformation online.

Parents in this study expressed emotional barriers when assessing health risks. Some experienced shame when their adolescents faced challenges such as mental health issues or social exclusion. Eaton et al. [37] discovered that parental self-stigma is linked to lower self-esteem and a reduced ability to take action. This internalized stigma may hinder objective assessment and delay needed intervention. Furthermore, while the parents expressed a desire to foster their adolescents’ independence, they questioned whether the adolescents themselves possessed adequate HL. Therefore, supporting adolescents in developing these competencies is both a parental responsibility and a protective strategy.

### Applying health information

Finally, HL includes the ability to apply health information to make evidence-based decisions related to one’s health [2]. This study demonstrated that parents struggle with the gap between knowing what is right (e.g., promoting sleep or a healthy diet) and influencing adolescent behavior. As one parent noted, “It’s one thing to know, another to pass it on” (P6). Our findings indicate that the legal and psychological shift in autonomy at age 16 is complicated, and it influences HL application. The parents were unsure of how to intervene without violating their adolescents’ privacy. This dilemma reflects broader challenges in parenting during late adolescence. Research by Branje [11] and Smetana and Rote [12] highlighted the importance of adaptive communication styles and boundary renegotiation in this phase.

For parents to effectively apply health information, this study suggests a need for more structured parental support, particularly in areas such as the impact of social media, societal pressures, and digital literacy. These needs align with findings from Jones et al. [32], who also identified communication and conflict management as particularly important topics for parental guidance. In light of Nutbeam’s HL model [4], our findings indicate that parents’ ability to access, interpret, and act on adolescent health information involves not only basic comprehension but also the deeper capacities for empowerment and for navigating structural barriers to support their adolescents’ health.

Another important insight from this study is that parenting experiences and strategies employed during early childhood influence parents’ current HL applications. It seems that parents who had worked to establish trust and open communication during earlier childhood phases felt more confident navigating the adolescent transition. This concurs with literature indicating that authoritative parenting fosters resilience and responsible decision-making in adolescents [38, 39]. However, our results indicate that the reduction of social venues for parental dialogue during adolescence, such as school meetings, limits opportunities for parents to share experiences and normalize challenges—a process considered easier when their children were younger. Therefore, strengthening parent networks, whether in person or digital, may help mitigate the isolating aspects of parenting during adolescence.

### Strengths and limitations

We employed Lincoln and Guba’s [40] criteria to ensure the credibility and trustworthiness of our findings, providing detailed descriptions of the analysis process. Participants’ quotations are included consistently to offer transparency and enrich the understanding of parental roles and experiences. Dependability was maintained by using a uniform interview guide inspired by the Conversational Health Literacy Assessment Tool, designed to assess HL during conversations [29], ensuring consistency in the questions posed to all participants. These efforts facilitated smooth conversation flow with participants offering detailed descriptions of their experiences. After ten interviews, we observed that participants were articulating predominantly similar, meaningful thematic issues. We conducted 15 interviews in total and judged data adequacy. We believe our findings are likely to be applicable to many parents of late-stage adolescents and may also be informative for professionals who support these families. However, transferability should be assessed in light of the study context and sample characteristics.

Despite these strengths, limitations of the present study exist. Conducting interviews via Zoom might have hindered spontaneity and restricted the capture of nonverbal cues, which are critical for comprehending participants’ reactions. Overall, the participant group is characterized by a predominance of higher education and active occupational engagement, with a small subset on disability benefits, providing a varied perspective for exploring HL among parents of late-stage adolescents. However, a high sociodemographic status among most participants suggests strong cognitive and social skills that may enhance their HL. Furthermore, the study includes only one father. These factors could shape the perspectives and experiences regarding HL and assessing and utilizing health information. This is important to consider when interpreting the results.

### Implications

Our findings indicate a need for targeted, feasible interventions aligned with parents’ needs that can empower parents’ confidence and skills to access, understand, appraise and apply reliable health information. Such interventions may improve parent–adolescent communication, support better health decisions, and promote positive adolescent health behaviors. To strengthen parental health literacy, we recommend practical strategies such as simple checklists or conversation guides for common adolescent topics (e.g., sexual health, mental wellbeing, substance use) that list key questions and trustworthy information sources. We also suggest school‑based, in‑person or virtual sessions timed to key transitions (e.g., entry to upper secondary) that explain adolescent health and how to access health services. Further research should examine HL from both parents’ and adolescents’ perspectives and specifically strive to include fathers of late-stage adolescents and parents with lower sociodemographic status.

## Conclusion

The findings indicate that parents of late-stage adolescents are committed to supporting their children in managing health-related issues throughout adolescence, highlighting the importance of parental HL for addressing both immediate health concerns and the development of adolescents’ long-term capacity for autonomous health management. However, many parents reported that losing access to their child’s health information after age 16 makes it harder to provide support. By facilitating the development of critical appraisal skills, and guiding informed decision-making, parents may contribute to strengthening adolescents’ competence and confidence in handling health-related matters more safely and independently. Empowering parents through strengthening their HL may improve individual and family health outcomes.

## Supplementary Information

Supplementary Material 1.

## Data Availability

Not appliccable (this manuscript does not report data generation or analysis).

## Abbreviations

HL: health literacy
