# Supplementary material for: Parental health literacy in parents of adolescents aged 16–19: a qualitative study
Source: BMC Public Health. 2026 Jan 31;26:758. doi: 10.1186/s12889-026-26471-9 (PMC12947484; doi:10.1186/s12889-026-26471-9)
Supplement: Supplementary file 1 — Supplementary Material 1. [file 12889_2026_26471_MOESM1_ESM.pdf]

Interview guide

Introduction

- Introduction of the researcher.
- Presentation of the research project and the purpose/aim of the project
- Parent introduce himself/herself
    - Age, current living situation, education level, employment status.

Parental role

- How do you experience life with teenage children? Elaborate.
    - Are there challenges? Elaborate.
- If you had the opportunity for guidance on being a parent to an adolescent, which topics would you like to focus on?

Health Literacy

The researcher briefly defines health literacy and why it is of interest.

- Do you perceive having sufficient information about health to care for your child's health, in relation to diet, sleep, medication use, mental health, etc.?
- How do you usually obtain information concerning health and illness? Where do you find information?
- How understandable is the information regarding your child's health?
    - E.g.: Vaccination, when to stay home from school, sick leave, menstruation
- On a scale from 0 to 10 (where 0 is poor and 10 is excellent), to what extent can you assess the credibility of health information?
- Do you feel confident in the decisions you make regarding your child's health?
- When your child turns 16, your access to health-related information about them becomes limited. Your child gains more responsibility for their own health, including consenting to treatment. Do you recall being informed about this transition as a parent?
    - If yes: Where/from whom did you receive the information? What were you informed about?
    - If no: Have you missed receiving information about this?
    - How do you experience transferring responsibility for health-related decisions to your adolescent?

    - What information would be beneficial for parents to receive regarding the increased health responsibility children gain upon turning 16?
- Have you experienced that your child has faced health challenges during their adolescence? Please provide an example (elaborate).
    - Who do you contact when your child faces health challenges?
    - How do you find healthcare professionals' communication and information provision?
    - On a scale from 0 to 10 (where 0 is poor and 10 is excellent), how comprehensible do you find the health information provided?
    - Did you feel understood and supported by healthcare professionals when your child was ill?
    - Is there anything you believe is particularly important for us to understand and consider when developing and tailoring health information for parents of adolescents?

Quality of Life

The researcher briefly defines quality of life and why it is of interest.

- What is important for you to have good quality of life?
- How do you assess your quality of life?
- Do you feel that your health affects your quality of life? If YES, elaborate.
- How does being a parent to an adolescent affect your quality of life?
- What do you do to ensure your own quality of life? (Resilience)

Conclusion

- Do you have any further thoughts or experiences related to these topics that you would like to share?
- How did you experience the interview?
